# Structural Performance of HDPE and WPC Lumber Components Used in Aquacultural Geodesic Spherical Cages

**DOI:** 10.3390/polym12010026

**Published:** 2019-12-21

**Authors:** Murtada Abass A. Alrubaie, Douglas J. Gardner, Roberto A. Lopez-Anido

**Affiliations:** 1 Department of Civil and Environmental Engineering, Advanced Structures and Composites Center, University of Maine, Orono, ME 04469, USA; rla@maine.edu; 2 School of Forest Resources, Advanced Structures and Composites Center, University of Maine, Orono, ME 04469, USA; douglasg@maine.edu

**Keywords:** buckling, WPC, HDPE, Southwell’s method, finite element analysis, Abaqus, aquacultural, structural analysis, wood, plastic, composite

## Abstract

Based on previous research, a novel wood–plastic composite (WPC) lumber has shown potential to replace high-density polyethylene (HDPE) lumber in the construction of aquacultural geodesic spherical cage structures. Six HDPE and six WPC assemblies, which are representative of typical full-size cage dimensions, were fabricated by bolting pairs of triangular panel components made with connected struts. Half of the panel assemblies had a plastic-coated steel wire mesh to simulate the actual restraint in field applications of the cages. The objective of the research was to characterize the structural performance of the panel assemblies under compressive loading. To determine the critical buckling load for the panel assemblies made from WPC and HDPE struts with and without wire mesh, Southwell’s method was implemented. A two-dimensional (2D) linear finite element analysis model was developed to determine axial forces in the struts of the panel assembly for the applied load and boundary conditions. This model was used to determine strut compressive forces corresponding to the Southwell’s method buckling load and the experimental failure load. It was found that the wire mesh increased the load capacity of both HDPE and WPC panel assemblies by a factor of two. The typical failure mode of the panels made from HDPE lumber struts, with and without wire mesh, was buckling of the struts, whereas the failure mode of the WPC panels, with and without wire mesh, was fracture at the notched section corresponding to the location of the bolts. The load capacity of the panel assemblies made from WPC lumber struts was three times and 2.5 times higher than the load capacity of the panel assemblies made from HDPE lumber struts with and without wire mesh, respectively.

## 1. Introduction

**Figure 1:**
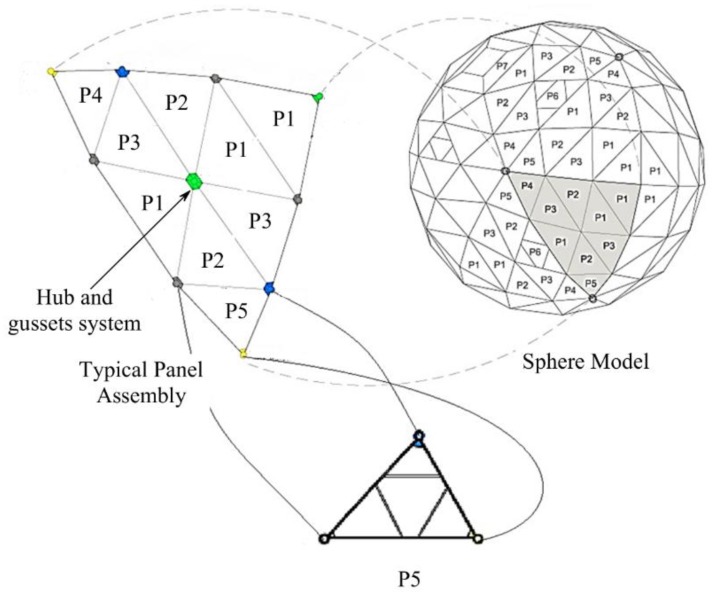
Details of connected struts in the panels and the connected panels to form the cage faces, types of hubs, and the types of the panels of the geodesic spherical cage structure with an approximate diameter of 21 m [1,2].

Aquaculture cages for fish farming are made in different ways. Unlike other types of aquaculture fish cage structures, the Aquapod Net Pen cage is a rigid-frame geodesic spherical cage structure. The cage structure is comprised of individual triangular panels made from high-density polyethylene (HDPE) lumber (strut), and these panels are fastened to each other to form the geodesic spherical shape of the cage [1]. These triangular panels are covered with wire mesh netting, which is affixed to the struts of the panels by mechanical fastening (stapling). Five panels (P1, P2, P3, P4, and P5) are the main structural components to construct the spherical shape of the Aquapod cage, as shown in Figure 1. These triangular panel components are designed to contribute to facile construction by reducing the time and manpower required to construct the cage structure. The cage structure is utilized in a fully submerged situation; except for cleaning, where it will be partially (30%) exposed to the air [1].

Because of the increased demand for aquaculture structures that have useful features (high volume capacity, rigid frame, and durable structure for up to 10 years) compared with other aquaculture cage structures, the geodesic spherical cage structure has been constructed in different volume capacities and diameters since 2006 [3] to its most recent product, Aquapod 4700, with a volume capacity of 4700 m^3^ (dia. of approximately 21 m) [3]. The cages function under submersion without any apparent problems from the marine exposure. However, damage to cage structures was reported in the Gulf of Mexico in 2015 [4], when the structures were exposed during cleaning to destructive surface waves during a hurricane. InnovaSea Systems, Inc. decided to explore a better material option to replace the HDPE lumber (struts). An extruded wood–plastic composite (WPC) lumber made from high-strength styrenic copolymer and thermally modified wood flour appears to be a promising alternative to replace HDPE, attributable to its desirable mechanical properties compared with HDPE lumber. For instance, the elastic modulus of the WPC lumber is approximately five time the elastic modulus of the HDPE lumber. Although WPCs have been investigated for structural applications [5,6,7,8,9,10,11,12], the performance of WPCs requires evaluation to be utilized for marine applications, where the material will be exposed to the combined effect of temperature and saltwater immersion. Nevertheless, it is very difficult to evaluate the structural performance of the full-scale structure of the cage structure that is made from HDPE or WPC lumber in such combined conditions (temperature and water immersion). WPCs are similar to other thermoplastic materials that exhibit viscoelastic behavior, hence their time-dependent behavior was investigated. Previous studies have focused on the time-dependent behavior of WPCs [5,13,14,15,16,17]. For the WPC lumber that is considered as an alternative to HDPE lumber in the construction of the cage structure, Alrubaie et al. conducted a 180-day creep experiment to compare the time-dependent behavior of HDPE and WPC lumber under similar conditions (temperature 23 ± 2 °C and relative humidity 50% ± 5%). Furthermore, the short-term time-dependent behavior of the WPC lumber (that is considered an alternative for the HDPE in the construction of the aquacultural geodesic spherical cage structure) was investigated and modeled under the synergistic effect of elevated temperature and water immersion [18,19]. InnovaSea Systems, Inc. conducted mechanical testing at the Advanced Manufacturing Center, University of Maine, Orono in 2006 to evaluate the buckling capacity of the full-scale fastened panels (with and without netting) of Aquapod A4700 made from glass bar-reinforced HDPE lumber.

The objective of the research presented here was to experimentally investigate and characterize the buckling capacity of two connected panels made from WPC and HDPE struts, with and without metallic mesh, to compare the structural performance of WPC lumber in aquacultural panel structures.

In this study, 24 triangular panels with struts length: 965, 1003, and 1321 mm were tested in compression along the longest strut. Twelve panels were made from WPC struts and twelve panels were made from HDPE struts. Six of each of these panels were constructed with plastic-coated steel wire mesh with 38.1 mm openings and 2.8 mm thickness of the steel wire [20]. A set of two panels were connected using three steel galvanized bolts with a diameter of 12.7 mm and two steel-galvanized square washers with dimension of 51 mm to each bolt. Four types of panels were experimentally investigated in the buckling experiment: WPC panels without the steel mesh condition (WPC-panel), WPC panels with the steel mesh condition (WPC-M-panel), HDPE panels without steel mesh (HDPE-panel), and HDPE panels with steel mesh (HDPE-M-panel). Three sets were tested for each panel type [21]. 

## 2. Experimental

### 2.1. Materials

**Figure 2:**
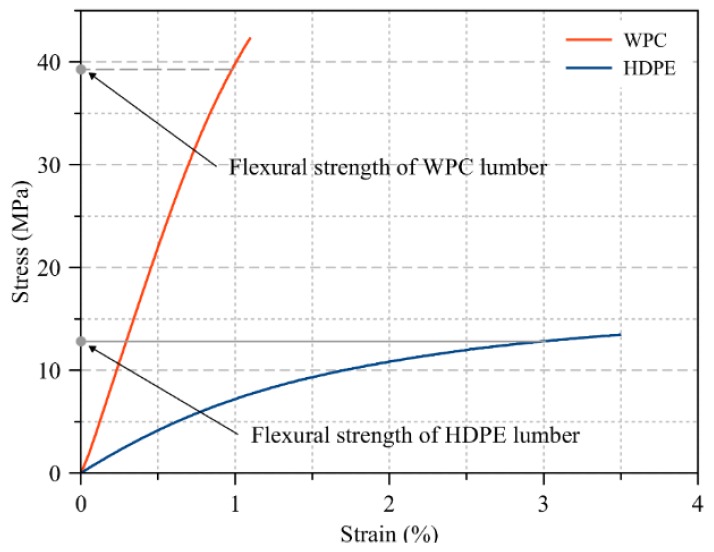
Typical stress versus strain relationship to obtain the elastic modulus and the flexural strength of WPC and HDPE lumber in accordance with ASTM D6109.

**Figure 3:**
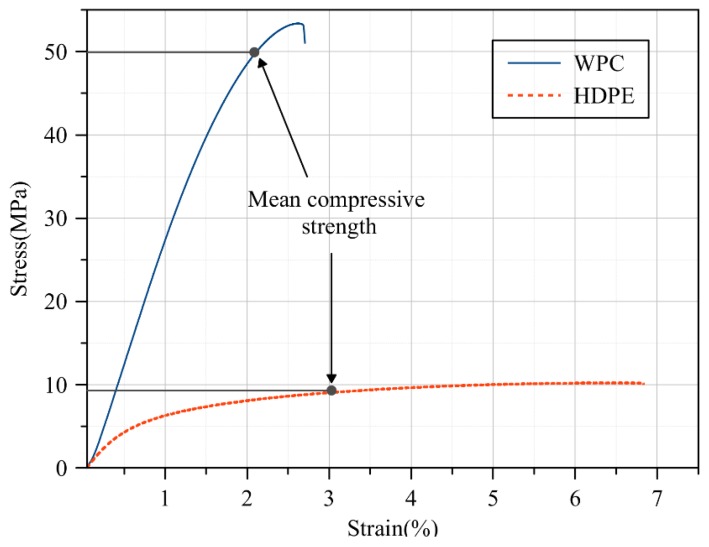
Typical stress versus strain relationship to obtain the elastic modulus and the compressive strength of WPC and HDPE lumber in accordance with ASTM D6108.

The commercially available HDPE lumber with cross section dimensions (b = 140 mm and h = 38.1 mm) was provided by InnovaSea Systems Inc. (Morrill, Maine, USA) and used in the manufacture of the HDPE triangular panels with and without steel wire mesh. The struts (made from WPC and HDPE lumber) of the triangular panels were connected to each other to form the panel via a triangular blocks (gussets) made from the same HDPE of the struts. The WPC lumber with cross section dimensions *b* = 139 mm and *h* = 33.5 mm was produced using a twin-screw Davis-Standard Woodtruder^TM^ (Orono, Maine, USA) in the Advanced Structures and Composites Center at the University of Maine’s Orono campus and were used in the manufacture of the WPC triangular panels with and without steel wire mesh. The WPC lumber examined is based on a patent-pending formulation that combines a thermally modified wood flour that was produced at a sawmill in Uimaharju, Finland and a high-strength styrenic copolymer system in an equivalent weight ratio to each of the two constituents. Flexural and compression tests were conducted to obtain the flexural and compressive properties of the WPC and HDPE lumber. Modulus of elasticity, flexural strength, and compressive strength of both materials were the properties obtained from these two tests and reported in Table 1. The number of samples was five samples for each test (flexure and compression). The length of the samples (*L*) of WPC and HDPE lumber tested in flexure was 545 and 620 mm to be in agreement with the required length (*L*) to depth (*h*) ratio in ASTM D6109 to be 16. Similarly, the length (*L*) of the WPC and HDPE lumber samples tested in compression was 160 and 183 mm to be in agreement with the ASTM D198 [22] of having the ratio of length (*L*) to the radius of gyration (*r*) to be less than 17. Prior to the testing of the WPC and HDPE lumber, the samples were conditioned (temperature 23 ± 2 °C and relative humidity 50% ± 5%) in accordance with ASTM D618 [23] for 96 h and tested under the same conditions in a climate-controlled mechanical laboratory at the Advanced Structures and Composites Center at the University of Maine, Orono, Maine. Figure 2 and Figure 3 show representative specimens of WPC and HDPE lumber tested in accordance with ASTM D6109 and ASTM D6108 [24] to obtain the modulus of elasticity, and the flexural and compressive strength, respectively. Unlike the WPC lumber, HDPE lumber did not exhibit failure in either the flexure or compression test. Thus, the flexural and compressive strengths were selected as the stress values that corresponded to the 3% strain in the stress–strain relationship in accordance with ASTM D6109 [24] and ASTM D6108 [25], respectively. 

### 2.2. Equipment and Test Setup

**Figure 4:**
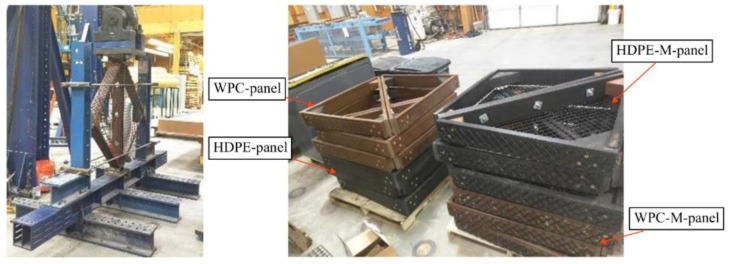
(**Left**) the test frame and the buckling test setup, (**right**) the WPC- and HDPE-connected triangular panels with and without metallic mesh.

The panel manufacture was conducted by InnovaSea Systems, Inc. The fixture to test the connected panels in compression was manufactured at the Advanced Structures and Composite Center, University of Maine, Orono, Maine. Figure 4 shows the connected triangular panels and the test fixture. An Instron (Norwood, MA, USA) test frame with a load cell capacity of 1334 kN was used. The data acquisition system (DAQ) with a written labview software was used to collect: the applied axial load (negative *Y*-direction in Figure 4), the axial displacement (of the actuator, the in-plane displacement of the struts), the in-plane displacement (negative and positive direction of *X*-axis as shown in Figure 4), and the out-of-plane displacement (negative and positive direction of *Z*-axis as shown in Figure 4). The tests were conducted in displacement control with a crosshead speed of 1 mm/min.

### 2.3. Degrees of Freedom of the Supports System of the Triangular Panels

**Figure 5:**
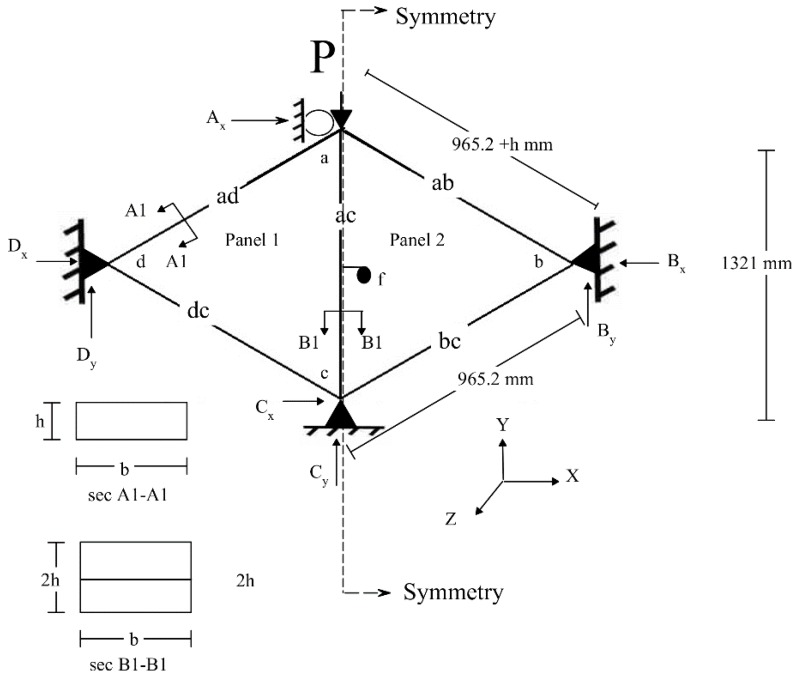
Schematic of the two-dimensional (2D) free body diagram of the tested connected (bolted) WPC and HDPE triangular panels with and without mesh.

The two-dimensional (2D) free body diagram shows the degree of freedom at each support as shown in Figure 5 and Table 2.

## 3. Results and Discussion

**Figure 6:**
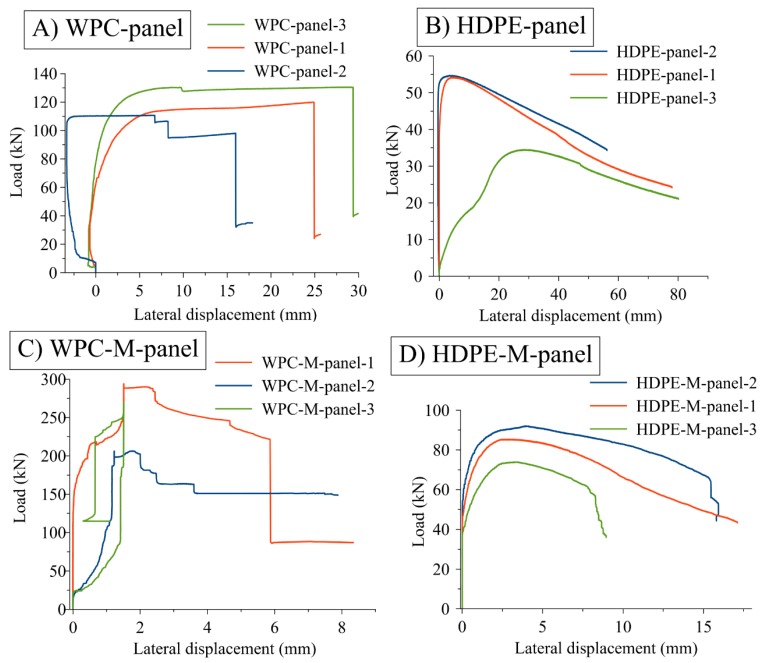
The relationship between the applied buckling load and lateral mid-span deflection of the vertical strut *ac* in the panels made from WPC and HDPE struts, and with and without metallic mesh.

The relationship between the applied buckling load and lateral deflection of the middle vertical strut *ac* at point *f* (Figure 5) for the connected components (panels) made from WPC and HDPE struts are reported in Figure 6. The buckling capacity of the panels made from WPC struts was three times the buckling capacity of the panels made from HDPE struts. The steel wire mesh contributed to the increased buckling capacity of the panels. A 2D finite element (FE) analysis model provided a useful assessment of the multiplier factor (α) that can be used in the computation of the reactions and the member of forces under different values of applied loads. Table 3 reports the values of α. Table 4 reports the average maximum buckling load of the HDPE and WPC strut-connected panels at each condition (with and without metallic mesh) and their corresponding type of failure.

The buckling failure of the cage components (panels) made from WPC struts was the dominant type of failure at one of the regions of the galvanized bolts that connect the two panels, causing a net section failure at the region where the bolts were located, whereas, no such net section failure was noticed at the buckling failure occurred for the panels made from HDPE lumber. This is attributable to the brittle behavior of the high wood-flour content WPC lumber compared with the HDPE plastic lumber. Table 4 summarizes the types of failure of the structural components of the cage structure with and without metallic mesh. Table 5 summarizes the implementation of the multiplier load factor (α) to compute the allowable member force of strut *ac* based on the buckling load values obtained from Southwell’s method [26]. Southwell’s method can be summarized by creating a plot based on the relationship between: the ratio of the lateral displacement (deflection) (Δ) over the applied buckling load (*P*), and the lateral displacement (Δ). If this relationship can be described by a linear relationship, then the inverse of the slope of this line represents the critical buckling load (*P*_cr_) and the buckling mode is global. This critical load does not account for imperfections or mode of interactions. 

Regarding the connected components (panels) made from WPC lumber (strut) without metallic mesh, the buckling failure tended to be abrupt after reaching the maximum applied load, as shown in Figure 6A. A similar pattern of the failure propagation was observed in the panels made from HDPE lumber without metallic mesh, the panels showed propagated deformation after reaching the maximum applied load without an abrupt failure, as shown in Figure 6B. However, panel number three (HDPE-panel-3), as shown in Figure 6B, exhibited a different load-lateral deflection curve. The panels (the middle strut *ac*) started deforming with the propagation of the applied load. This can be attributed to the geometry of the panels or to an eccentricity that developed while the load was imposed to the panel. 

**Figure 7:**
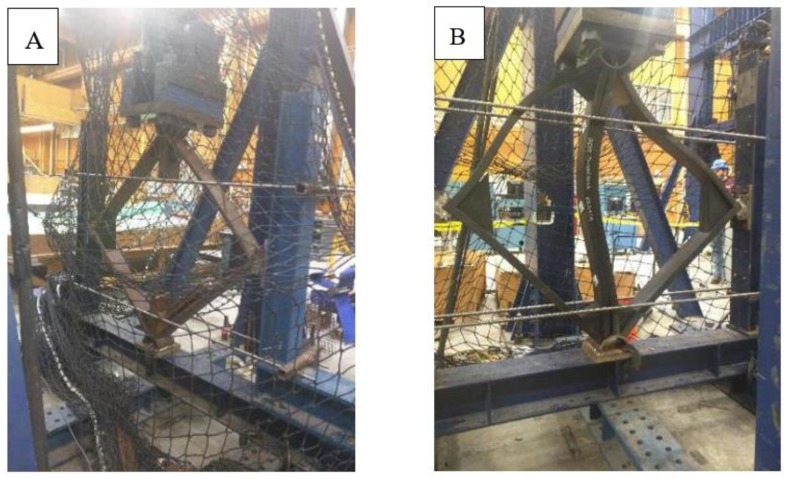

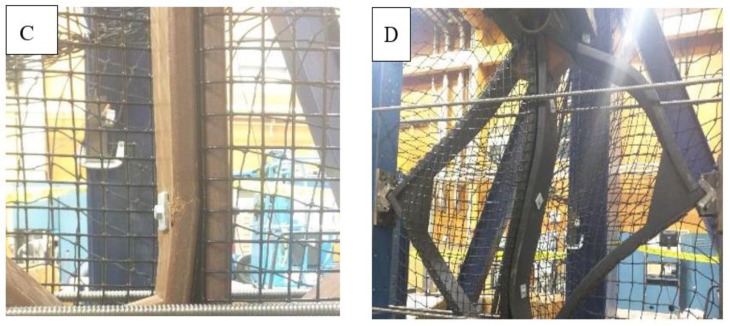
Failure modes of the panels made from HDPE and WPC lumber for the four different cases; (**A**) net section failure at the middle strut *ac* at the location of the bolt connection of the panels made from WPC without metallic mesh, (**B**) buckling mode failure of the strut *ac* of the panels made from HDPE without metallic mesh, (**C**) net section failure mode of panels made from WPC struts with metallic mesh, and (**D**) buckling failure mode of the panels made from HDPE struts with metallic mesh.

Regarding the failure behavior of the panels made from WPC with metallic mesh, the metallic mesh contributed to an increase in the buckling capacity (maximum applied load) approximately three times of the buckling capacity (maximum applied load) of the panels made without the metallic mesh. However, as regards to improving the ductility of the panels, the metallic mesh did not contribute to improving the ductility of the panels made from WPC struts. The panels showed a lateral deformation smaller than 2 mm before reaching the maximum applied load and then experiencing abrupt failure. Moreover, the failure mode (of the panels made from WPC struts with mesh) in the struts did not change from the failure mode of the panels without the metallic mesh, which is the net section failure at the connected struts attributable to the buckling in the *X*-axis at the strut *ac* (Figure 6C). Figure 7 shows the failure modes of the panels made from HDPE and WPC lumber for the four different cases. 

The metallic mesh is affixed to the WPC and HDPE struts by staples on the perimeter of the struts (on the width b of the cross section of the struts (sections A1-A1 and B1-B1) in Figure 5). The staples have shown good resistance to the applied load and in developing the buckling capacity of the panels with the metallic mesh. This can be observed with the small lateral displacement of the panels (with metallic mesh) made from WPC and HDPE struts of 1.5 and 3.4 mm, corresponding to the maximum buckling capacity, respectively. Whereas, the lateral displacements for the same type of panels without metallic mesh were 20.1 and 12.5 mm, respectively.

### 3.1. Structural Analysis of the Tested Structural Components (Panels) of the Geodesic Spherical Cage Structure

To compute the reaction and the section forces at the supports and the struts of the connected, respectively, 2D (two-dimensional) finite element (FE) linear elastic analyses models were conducted to the four types of the test panels (WPC-M-panel, WPC-panel, HDPE-M-panel, and HDPE-panel) using commercially available software Abaqus/CAE with the following assumptions:

1. Based on the symmetry of the connected panels, panel 1 in Figure 5 was used on the 2D FE model to compute the member forces and the support reactions. 

2. The supports at points b, c, and d were assumed to be as pin supports (vertical (*Y*-axis) and horizontal (*X*-axis) movement restriction), whereas, point a was assumed to be as s roller support (horizontal (*X*-axis) movement restriction). This assumption was made based on the design of the fixture used in the experiment and the ability of the structure to have rotation at the points a, b, c, and d. 

3. A slender beam element B23 (cubic beam in plane) was chosen from the available types of beam elements available in the used commercial software Abaqus and was used in the 2D linear finite element (FE) analysis of the structural components of the geodesic spherical cage structure. The selection was made based on the assumption that both the struts of the components and the metallic mesh are slender even some of the beams have a slender ratio (span (l)/ radius of gyration (r)) less than 200. This assumption eliminated the need to have the values of Poisson’s ratio of the materials of the components (steel of the metallic mesh, WPC struts, and HDPE struts), i.e., the elastic moduli were the required input for the mechanical properties of the materials in the 2D FE linear analysis model.

4. Regarding the connected panels with metallic mesh, the metallic mesh was modeled as vertical and horizontal beam elements [type B23] (each wire mesh modeled as a beam) spaced 38.1 mm from each other and has a circular cross-section with a diameter of 3 mm to each beam. The geometry and the space of the wire mesh was implemented based on the specification of the metallic wire mesh, Aquamesh^®^, used in the manufacture of the structural panels of the geodesic spherical cage structure.

5. The elastic moduli of the WPC and HDPE lumber used in the structural analysis were obtained from the 4-point bending test conducted on specimens with a span to depth ratio of 16 to be 4430 and 930 MPa as reported in Table 1, respectively. The elastic modulus of the wire mesh was assumed to be the elastic modulus of steel, *E*_steel_ = 200 GPa.

6. The 2D FE model was conducted to investigate the response of the structure in the linear region. Thus, the values of the applied load were assumed to be a unit load (1 N) to be applied to the structure. The computed member forces and reactions at the supports represented a multiplier coefficient that can be used to compute the reactions and member forces at any value of the applied load. 

**Figure 8:**
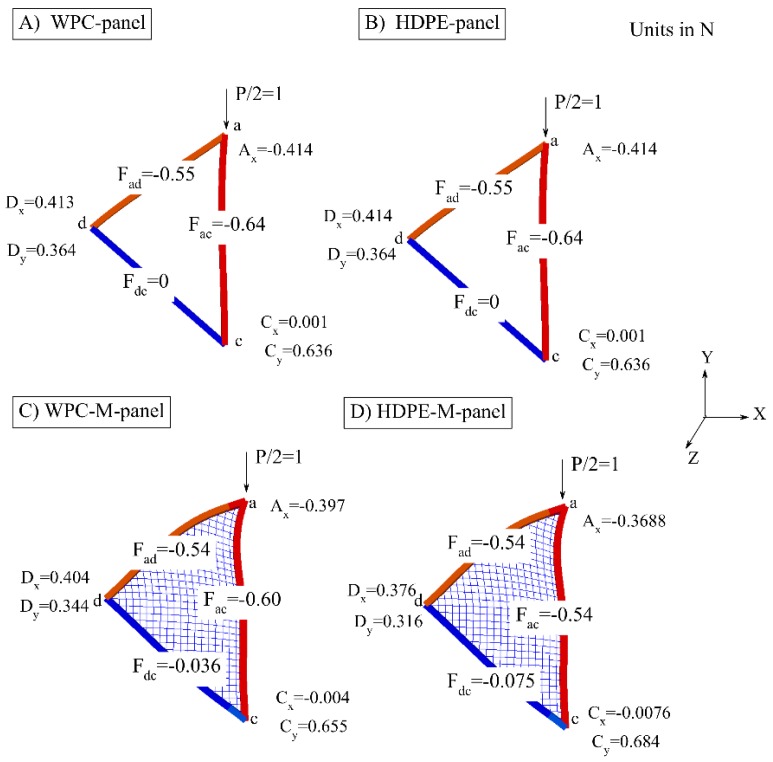
Reactions and member forces in N units of panel 1 of the geodesic spherical cage structure obtained from 2D FE linear analyses; (**A**) WPC-panel, (**B**) HDPE-panel, (**C**)WPC-M-panel, and (**D**) HDPE-M-panel.

The reaction values at the supports were computed from the 2D FE model. Furthermore, the member forces were computed for the tested panels in the four cases, to provide an understanding to the distribution of the applied load through the struts of the panels. However, the strut *cd* for the panels made from HDPE or WPC lumber without metallic mesh had no member force. Whereas, the metallic mesh contributed into distributing the applied load among the struts; *ac*, *cd*, and *ad*. Furthermore, the value of the member force varied along the length of the strut attributable to the presence of the metallic mesh. The maximum values of member forces of the struts of the panels made from HDPE and WPC struts with metallic mesh are shown and reported in Table 3 and Figure 8, respectively. 

### 3.2. Implementation of Southwell’s Method to Determine the Critical Load

**Figure 9:**
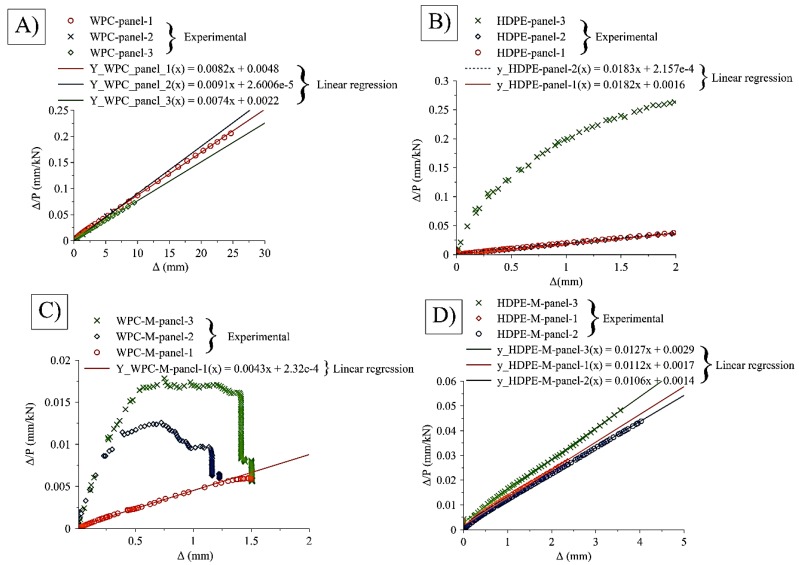
Application of Southwell’s method to obtain the critical buckling load of the structural panels of the cage structure made from: (**A**) WPC-panel, (**B**) HDPE-panel, (**C**) WPC-M-panel, and (**D**) HDPE-M-panel.

To investigate the critical load mode for the four cases of the panels made from WPC and HDPE struts in the cases of metallic wire mesh and without wire mesh, Southwell’s method was implemented. The method was implemented on the relationships reported in Figure 9 after modifying the relationship to include the load vs later deflection only at the limit of the maximum applied load (i.e., the data points after the maximum applied load has not been considered in the application of Southwell’s method). By using linear regression to obtain the slope of the equation of the line, hence, the inverse of the slope of the line represents the value of *P*_cr_. Figure 9 shows the implication of Southwell’s method on the four sample panels (WPC-panel, WPC-M-panel, HDPE-panel, and HDPE-M-panel) and the obtained slope of each tested set of panels. Based on the linear relationship between Δ/P versus Δ, the critical load can be determined. 

## 4. Conclusions

1. The buckling behavior of the structural components of the geodesic spherical cage structure made from HDPE and WPC lumber was experimentally investigated and characterized. The buckling capacity (load) of the triangular panels made from WPC struts and with mesh was 256.81 kN, whereas the buckling capacity of the same type of panels made from HDPE struts was 83.80 kN. Furthermore, the buckling capacity of the panels made from WPC struts and without steel mesh was 120.42 kN, which was 2.5 times the buckling capacity of the same condition of panels but made from HDPE struts. 

2. The metallic mesh contributed into distributing the member forces through the struts: *ac*, *ca* and *da* of the component (panel). Whereas, the panels without metallic mesh experienced strut (*cd*) without member force (Figure 8). 

3. The structural analyses conducted on the triangular components (panels) of the geodesic spherical cage structure, the compression, and flexure tests have shown that the failure occurrence in the triangular components (panels) was attributable to bending in the struts (Table 1).

4. Attributable to the brittleness behavior of WPC lumber (50 wt. % wood flour) compared with the ductile behavior of HDPE lumber (100 wt. % plastic), an abrupt failure to panels made from WPC was observed in the experiments.

5. According to the linear structural analysis, the short struts in the connected panels (struts *bc* and strut *cd*) did not carry load values and this contributed to the buckling occurrence to be initiated in the longest strut (*ac*) and then at the shorter struts (*ab* and *ad*) for the panels made from WPC and HDPE lumber and without metallic mesh. 

6. As a containment aquaculture structure system in open ocean environments, it is preferable to have a structural material (strut) that shows an indication prior to failure, or to defect without breakage, than an abrupt failure, so that the member can be replaced properly.

7. Attributable to the viscoelastic behavior and the brittleness behavior of the WPC in this study, it is preferable to consider using the WPC lumber in structural applications where the applied load should be at a low level compared with strength of the WPC, to avoid the abrupt failure of the structural member during the service life of the structure. 

8. The finite element analyses (Figure 8) conducted in this study by applying a unit load considered a useful tool that can be used to compute the reactions and the member forces in the struts of a similar test setup subjected to different values of loading. This analyses also help to compute the reactions and the member forces in the struts for similar test setup panels but in a different scale.

9. The findings of this study that the loading capacity of the connected panels with metallic mesh is twice the loading capacity of the panels made without metallic mesh for both HDPE and WPC struts, is considered a powerful tool to minimize the computational efforts in the design and the analysis of similar structures with and without metallic mesh. Thus, the structures can be analyzed by ignoring the metallic mesh and then can be multiplied by a factor of two to consider the effect of the metallic mesh.

## Figures and Tables

**Table 1:** Mechanical properties of wood–plastic composite (WPC) and high-density polyethylene (HDPE) lumber used as struts of the geodesic components of the Aquapod net pen geodesic spherical cage structure.

Type of Test	Obtained Properties	WPC	HDPE
Flexure (ASTM D6109)	Modulus of elasticity (E)/GPa	4.3 ± 0.3	0.9 ± 0.03
	Modulus of rupture (strength)/MPa	41.2 ± 4.5	14.1 ± 0.7
Compression (ASTM D6108)	Modulus of elasticity (E)/GPa	4.3 ± 1.0	0.8 ± 0.1
	Strength/MPa	50.8 ± 1.9	8.8 ± 0.1

**Table 2:** Two-dimensional degrees of freedom of the triangular panels during the buckling experiment.

Supports Boundary Conditions (Free = 0, Fixed = 1)	Supports			
	*a*	*b*	*c*	*d*
U_x_	1	1	1	1
U_y_	0	1	1	1
θ_z_	0	0	0	0

**Table 3:** Reactions and member forces computed from the 2D finite element (FE) linear analyses obtained from applying unit load on panel 1 (Figure 5 at point a) of the geodesic spherical cage structure for four sample types: WPC with steel mesh (WPC-M)-panel, WPC-panel, HDPE with steel mesh (HDPE-M)-panel, and HDPE-panel.

Reactions and Member Forces (N)	Condition of the Panels Made from WPC Struts		Condition of the Panels Made from HDPE Struts	
	M-Panel	Panel	M-Panel	Panel
**A_x_**	−0.4	−0.414	−0.37	−0.414
**C_x_**	−0.004	0.001	0.01	0.001
**C_y_**	0.66	0.64	0.7	0.64
**D_x_**	0.404	0.413	0.38	0.413
**D_y_**	0.34	0.36	0.32	0.36
**F_ad_**	−0.54	−0.55	−0.54	−0.55
**F_ac_**	−0.60	−0.64	−0.54	−0.64
**F_cd_**	−0.04	0	−0.1	0

**Table 4:** The experimental maximum buckling load and the failure type and occurrence sequence in the structural components of the geodesic spherical cage structure.

Connected Triangular Panels		Experimental Buckling Load for Each Panel (kN)			Failure Type and Location of Occurrence in the Component	
Struts Material	Mesh Condition M-panel = with Mesh Panel = without Mesh	P1	P2	P3	M-Panel	Panel
WPC	M-panel	294	207	270	Struts Buckling failure (X-axis) and metallic mesh buckling (Z-axis)	Buckling failure (X-axis)
WPC	Panel	120	111	131		
HDPE	M-panel	85	92	74	Metallic mesh buckling (Z-axis) followed by struts buckling (X-axis)	Buckling failure (X-axis) in the struts
HDPE	panel	54	55	35		

**Table 5:** The buckling load of the member (strut) *ac* based on multiplying the multiplier value α obtained from the 2D FE linear analyses by the value of critical load obtained from Southwell’s method.

Connected Panels	Experimental	Southwell’s Method	Euler’s Method	Allowable Buckling Load
	Max. Average Load (kN)		$\frac{4\pi^{2}EI}{l^{2}}$	
Material and Mesh Condition	*F*_ac_	*F*_ac-Southwell-critical_ (kN)	*F*_ac-Euler-critical_ (kN)	*F*_ac_ (kN)
WPC-M-panel	129	116	NA	70
WPC-panel	60	61	43	39
HDPE-M-panel	42	44	NA	24
HDPE-panel	24	27	14	17

## References

[1] Vandenbroucke K., Metzlaff M. (2013). Abiotic Stress Tolerant Crops: Genes, Pathways and Bottlenecks. Sustainable Food Production.

[2] Page S.H. (2013). Aquapod Systems for Sustainable Ocean Aquaculture. Sustainable Food Production.

[3] InnovaSea Systems, Inc. (2016). A4700 Bridle System in Grid Mooring Cell. https://www.innovasea.com.

[4] InnovaSea Systems, Inc. (2015). Report on Structural Damage to A4800 AquaPod.

[5] Alvarez-Valencia D., Dagher H.J., Davids W.G., Lopez-Aodio R.A., Gardner D.J. (2010). Structural performance of wood plastic composite sheet piling. J. Mater. Civ. Eng..

[6] Balma D.A. (1999). Evaluation of Bolted Connections in Wood Plastic Composites. Master’s Thesis.

[7] Brandt W.C., Fridley K.J. (2003). Load-duration behavior of wood-plastic composites. J. Mater. Civ. Eng..

[8] Brandt W.C., Fridley K.J. (2007). Effect of load rate on flexural properties of wood-plastic composites. Wood Fiber Sci..

[9] Dura M.J. (2005). Behavior of Hybrid Wood Plastic Composite-Fiber Reinforced Polymer Structural Members for Use in Sustained Loading Applications. Master’s Thesis.

[10] Haiar K.J. (2000). Performance and Design of Prototype Wood-Plastic Composite Sections. Master’s Thesis.

[11] Slaughter A.E. (2006). Design and Fatigue of a Structural Wood-Plastic Composite. Master’s Thesis.

[12] Tamrakar S. (2011). Effect of Strain Rate and Hygrothermal Environment in Wood Plastic Composite Sheet Piles. https://digitalcommons.library.umaine.edu/etd/1576.

[13] Chang F.-C. (2011). Creep Behaviour of Wood-Plastic Composites. Ph.D. Thesis.

[14] Hamel S.E. (2011). Modeling the Time-Dependent Flexural Response of Wood-Plastic Composite Materials. Ph.D. Thesis.

[15] Pooler D.J., Smith L.V. (2004). Nonlinear viscoelastic response of a wood–plastic composite including temperature effects. J. Thermoplast. Compos. Mater..

[16] Sandeep T., Lopez-Anido R.A., Kiziltas A., Gardner D.J. (2011). Time and temperature dependent response of a wood–polypropylene composite. Compos. Part A Appl. Sci. Manuf..

[17] Alrubaie M.A. (2019). Investigating the Time-dependent and the Mechanical Behavior of Wood Plastic Composite Lumber Made from Thermally Modified Wood in the Use of Marine Aquacultural Structures. https://digitalcommons.library.umaine.edu/etd/3026.

[18] Alrubaie M.A.A., Lopez-Anido R.A., Gardner D.J., Tajvidi M., Han Y. (2019). Experimental investigation of the hygrothermal creep strain of wood–plastic composite lumber made from thermally modified wood. J. Thermoplast. Compos. Mater..

[19] Alrubaie M.A.A., Lopez-Anido R.A., Gardner D.J., Tajvidi M., Han Y. (2019). Modeling the hygrothermal creep behavior of wood plastic composite (WPC) lumber made from thermally modified wood. J. Thermoplast. Compos. Mater..

[20] Caccese V. (2006). Structural Testing of Various Configurations for the AquaPod Net Pen.

[21] Tangent Technologies LLC (2015). Polyforce Sturctural Recycled Plastic Lumber. [Cited 2018 November; Mechanical Propertied of the HDPE Polyforce Lumber]. http://tangentusa.com/wp-content/uploads/2016/01/PolyForce_DataSheet_01_20_16.pdf.

[22] ASTM International (2015). Standard Test Methods of Static Tests of Lumber in Structural Sizes, D198-15.

[23] ASTM International (2013). Standard Practice for Conditioning Plastics for Testing, D618-13.

[24] ASTM International (2013). Standard Test Methods for Flexural Properties of Unreinforced and Reinforced Plastic Lumber and Related Products, D6109-13.

[25] ASTM International (2019). Standard Test Method for COmpressive Properties of Plastiv Lumber and Shapes, D6108-19.

[26] Barbero E., Tomblin J. (1993). Euler buckling of thin-walled composite columns. Thin Walled Struct..

